# Observational study on quality of life, safety, and effectiveness of first‐line cetuximab plus chemotherapy in *KRAS* wild‐type metastatic colorectal cancer patients: the ObservEr Study

**DOI:** 10.1002/cam4.888

**Published:** 2016-10-17

**Authors:** Carmine Pinto, Francesca Di Fabio, Gerardo Rosati, Ivan R. Lolli, Enzo M. Ruggeri, Libero Ciuffreda, Daris Ferrari, Giovanni Lo Re, Giovanni Rosti, Paolo Tralongo, Raimondo Ferrara, Oscar Alabiso, Silvana Chiara, Giovanni P. Ianniello, Antonio Frassoldati, Domenico Bilancia, Giovanna A. Campanella, Carlo Signorelli, Patrizia Racca, Elena Benincasa, Maria Elena Stroppolo, Francesco Di Costanzo

**Affiliations:** ^1^ Medical OncologySanta Maria Nuova IRCCS HospitalReggio EmiliaItaly; ^2^ Medical OncologyS. Orsola‐Malpighi Policlinic HospitalBolognaItaly; ^3^ Medical OncologySan Carlo HospitalPotenzaItaly; ^4^ Medical OncologySaverio de Bellis IRCCS HospitalCastellana GrotteItaly; ^5^ Medical OncologyBelcolle AUSL Hospital ViterboViterboItaly; ^6^ Medical OncologyCittà della Salute e della Scienzaand San Giovanni Battista ‐ Molinette HospitalsTurinItaly; ^7^ Medical OncologySan Paolo HospitalMilanItaly; ^8^ Medical OncologyPordenone AAS5 HospitalPordenoneItaly; ^9^ Medical OncologyTreviso Regional HospitalTrevisoItaly; ^10^ Medical OncologyUmberto I RAO HospitalSiracusaItaly; ^11^ Medical OncologyMons. R. Dimiccoli HospitalBarlettaItaly; ^12^ Medical OncologyCarità HospitalNovaraItaly; ^13^ Medical OncologySan Martino IRCCS University Hospital – National Cancer InstituteGenoaItaly; ^14^ Medical OncologySant'Anna e San Sebastiano HospitalCasertaItaly; ^15^ Clinical OncologyArcispedale Sant'Anna University HospitalFerraraItaly; ^16^ Merck Serono S.p.A.RomeItaly; ^17^ Careggi University HospitalFlorenceItaly

**Keywords:** Cetuximab, metastatic colorectal cancer, quality of life, skin reactions

## Abstract

Cetuximab improves efficacy when added to chemotherapy for metastatic colorectal cancer (mCRC). Effective management of skin reactions from cetuximab improves quality of life (QoL), and treatment compliance in clinical trials. No data are available from real‐world settings. The ObservEr observational, multicenter, prospective study evaluated QoL, the incidence of skin reactions, and management of chemotherapy plus cetuximab in first‐line for mCRC. The primary endpoint was QoL measured with the Dermatology Life Quality Index (DLQI) and EORTC QLQ‐C30. Secondary endpoints were the incidence of skin and serious adverse events, median overall and progression‐free survival, tumor response, and resection rates. Between May 2011 and November 2012, 228 patients with KRASwt mCRC were enrolled at 28 Italian centers, 225 evaluable, median age 65 years. QoL did not change during treatment and was not affected by the choice of prophylactic or reactive skin management. The incidence of cetuximab‐specific grade ≥3 skin reactions was 14%, with no grade 4/5 events. Skin reactions correlated with survival (*P* = 0.016), and their incidence was influenced by chemotherapy regimen (oxaliplatin vs. irinotecan—Incidence rate ratio [IRR] 1.72, *P* < 0.0001) and gender (male vs. female—IRR 1.38, *P* = 0.0008). Compliance at first postbaseline evaluation was 97.75%. Median overall survival was 23.6 months, median progression‐free survival 8.3 months. Cetuximab plus chemotherapy did not compromise QoL in the routine clinical setting when patients receive close monitoring plus prophylactic or reactive management of skin reactions. We observed the same correlation between overall survival (OS) and skin reactions reported in controlled clinical trials, also in this setting.

## Introduction

Based on results from phase II and III trials 1, 2, 3, 4, 5, and a pooled analysis 6, cetuximab is recommended in combination with standard first‐line treatment regimens for patients with RAS wild‐type metastatic colorectal cancer (mCRC) 7, 8. The efficacy of cetuximab combination therapy is positively correlated with severe skin reactions. Although these reactions do not appear to influence global health status (GHS), overall quality of life (QoL), or social functioning 9, they do adversely affect skin‐related QoL measured with the DLQI 10, and have a negative impact on therapy compliance 9, 11, 12. QoL is unquestionably a clinically relevant outcome; however, it is rarely measured in routine practice. Data from randomized trials of cetuximab show that effective monitoring and management of skin reactions improves QoL and treatment compliance. No data are available from real‐world settings.

The ObservEr study (noninterventional, observational, multicenter, prospective study of QoL, safety, and efficacy of first‐line chemotherapy in combination with cetuximab) is the first observational study of first‐line cetuximab combined with standard chemotherapy in Italian patients with wild‐type *KRAS* mCRC tumors. This study provides an overview of QoL, the incidence of serious skin reactions, and efficacy with cetuximab in the first‐line setting.

## Patients and Methods

The ObservEr study had competitive enrollment at 28 Italian centers (May 2011 through November 2012) of patients with *KRAS*‐wild‐type mCRC planning to receive first‐line treatment with cetuximab plus chemotherapy. The mCRC indication for cetuximab changed from *KRAS* to *RAS* wild type in December 2013, after enrollment had been completed 13. The protocol was approved by the independent ethics committee at each participating center and complied with International Ethical Guidelines for Biomedical Research Involving Human Subjects, Good Clinical Practice guidelines, the Declaration of Helsinki, and local laws on observational studies. All patients provided written informed consent.

### Patients

Main inclusion criteria were as follows: age ≥ 18 years; eligible to receive treatment with cetuximab plus chemotherapy (i.e., Eastern Cooperative Oncology Group performance status 0 or 1; 8); histologically proven and measurable (RECIST v1.1) metastatic adenocarcinoma of the colon or rectum; chemonaïve for metastatic disease; *KRAS* exon 2 wild‐type tumors; and planned cetuximab treatment according to the SmPC. Patients with prior investigational drug/agent/procedures were excluded. In each center, all consecutive eligible patients were prospectively enrolled in the study.

### Treatment

Cetuximab was administered weekly in association with chemotherapy. Patients were treated until disease progression or unacceptable toxicity, according to clinical practice at the center. Treatment compliance (%) was calculated as total doses received / total planned doses × 100. Before starting therapy, investigators defined how they would manage skin toxicity in each patient, selecting one of the three skin protocols: (1) prophylactic, (2) reactive, or (3) according to usual clinical practice at their center 14. Skin Protocol 1 was started 1 day before the first cetuximab dose and consisted of topical vitamin K1 (Vigorskin©, MERCK Serono S.p.A, Rome, Italy) for ≥ 8 weeks. Skin Protocol 2, for managing grade 2–4 emergent skin toxicity, consisted of topical vitamin K1 applied as in Protocol 1 combined with doxycycline 100 mg per os twice daily.

### Endpoints and measurement

The primary endpoint was QoL. Cetuximab‐related skin reactions generally develop within the first 3 weeks of therapy 12, thus measuring QoL within the first 8–12 weeks of therapy allowed assessment of the impact of skin reactions. Patient‐reported outcomes were evaluated in all treated patients who had completed the baseline assessment and at least one postbaseline assessment that included completing the DLQI 15 and EORTC Quality of Life Questionnaire (QLQ) C30 version 3.0 (EORTC DataCenter, Brussels). Patients completed the DLQI questionnaire at baseline and weekly during the first 8 weeks, then at every evaluation visit scheduled per local clinical practice until disease progression. EORTC QLQ‐C30 questionnaires were completed at baseline, first postbaseline evaluation (week: 8–12), and every subsequent evaluation visit.

Secondary endpoints were as follows: efficacy of the different skin management protocols assessed with the DLQI; incidence of cetuximab‐related skin toxicity and any serious AE (SAE); median overall survival (OS) and proportion of patients still alive at 2 years; progression‐free survival (PFS); overall response rate (ORR); metastases resection rate (mRR); and time required to receive *KRAS* laboratory test results. AEs were graded using National Cancer Institute Common Terminology Criteria for Adverse Events v4.03. OS was defined as months from first cetuximab dose to death or last contact when a death has not been registered; PFS was calculated as the time from start of therapy to evidence of clinical/radiologic progression. ORR was defined as the sum of complete responses (CR) and partial responses (PR). Both PFS and ORR were evaluated using RECIST v1.1 (Revised RECIST guideline (version 1.1). EJC 2009;45:228‐47). Radiologic assessment was per local clinical practice (every 8–12 weeks). Absence of a scheduled per protocol time (observational study) represents a potential source of bias for PFS and ORR. Time to *KRAS* test results were calculated as days between date of request and date of results.

### Statistical analysis

Descriptive summary statistics for continuous variables comprised number of nonmissing observations, mean, standard deviation (SD), median, lower and upper quartile, minimum, and maximum, where appropriate. Frequencies and percentages were provided for categorical variables. DLQI and EORTC QLQ‐C30 results according to skin protocol chosen at baseline were evaluated by both *t*‐tests and an ANCOVA model, while changes in total score from baseline to first postbaseline visit according to predictors (age: ≥75 years, primary tumor surgery at baseline, >1 metastatic site, irinotecan combination, best response in first‐line, skin toxicity in the first 8 weeks) were evaluated with an ANOVA model. The incidence of cetuximab‐related skin reactions according to age, chemotherapy (oxaliplatin vs. irinotecan), and gender were calculated with a multiple Poisson regression test. *P*‐values are reported and statistical significance declared for *P *<* *0.05, without correction for multiplicity. OS and PFS were analyzed using the Kaplan–Meier method. An exploratory landmark analysis of OS was conducted in subgroups defined by skin toxicity (any grade vs. no skin toxicity).

Sample size required to evaluate QoL was 210 patients, based on:

☐ The SD for changes in DLQI scores is approximately 7 units; 16 thus, if 80% of the 210 patients scheduled for enrollment (*n *=* *167) were evaluable for the analysis of change in DLQI scores from baseline, then the distance from the boundaries of the two‐sided 95% confidence interval (CI) to the point estimate would be 1.07 units.
☐ DLQI total scores ranging from 0 to 1 were interpreted as no effect on dermatology‐related QoL, from 2 to 5 as a small effect, 6 to 10 as a moderate effect, 11 to 20 as a very large effect, and 21 to 30 as an extremely large effect 15.

☐ The SD for changes in EORTC QLQ‐C30 GHS score is approximately 25 units; 17 thus, if 80% of the 210 patients scheduled for enrollment (*n *=* *167) were evaluable for the analysis of change in EORTC QLQ‐C30 GHS from baseline to week 8, then the distance from the boundaries of the two‐sided 95% CI to the point estimate would be 3.82 units.
☐ A 10‐unit difference in the change in scores was considered clinically important 18, 19.

## Results

**Figure 1:**
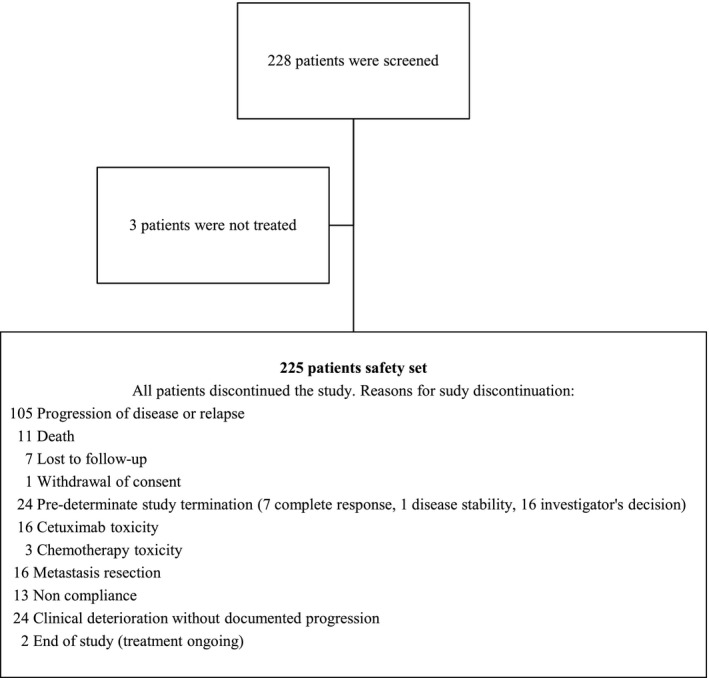
Patient disposition in the ObservEr Study.

Between May 2011 and November 2012, 228 patients were enrolled at 28 Italian centers; 225 were evaluable for safety analysis (Fig. 1). All patients in the safety population completed the study per protocol. Median age was 65 years. Demographic characteristics are summarized in Table 1.

**Table 1:** Demographic and baseline characteristics of the safety population (*N* = 225)

Characteristic	Patients
Age, years, median (range)	64.76 (39–81)
Age (class) *n* (%)	
<70 years	141 (62.2)
≥70 years	84 (37.3)
Gender, *n* (%)	
Male	149 (66.2)
Female	76 (33.8)
Body surface area, m^2^, mean (SD)	1.78 (0.192)
Ethnicity, *n* (%)	
White	223 (99.1)
Asian	1 (0.4)
Other	1 (0.4)
ECOG performance status, *n* (%)	
0	175 (76.8)
1	50 (23.2)
Any disease, MedDRA SOC v.15, *n* (%)	118 (52.4)
Primary tumor site, *n* (%)	
Colon/upper rectum	154 (68.4)
Median/lower rectum	71 (31.6)
Primary tumor surgery, *n* (%)	162 (72.0)
Patients with prior adjuvant chemotherapy, *n* (%)	80 (35.6)
Number of metastatic sites, *n* (%)	
1	141 (62.7)
>1	84 (37.3)
Site of metastasis, *n* (%)	
Liver	158 (70.2)
Only liver	97 (43.1)
Lung	53 (23.6)
Bone	4 (1.8)
Lymph node	64 (28.4)
Other	51 (22.7)

All 225 patients received cetuximab plus chemotherapy (Table 2), which was irinotecan‐based in 145 (64%), oxaliplatin‐based in 67 (30%), and other fluoropyrimidine‐based in 13 (6%). At baseline, prophylactic skin management (Protocol 1) was chosen for 160 patients (71.1%), reactive management (Protocol 2) was followed in 37 (16.4%), and a local institutional protocol (Protocol 3) was applied in the remaining 28 (12.4%). Compliance was 97.75% for the total population at week 8: compliance was ≥90% in 208 patients (92.4%), 70–89% in 15 (6.7%), and ≤69% in two (0.9%). After week 8, compliance was 96.8% for the 168 assessable patients. There was no difference in compliance between patients receiving irinotecan or oxaliplatin‐based chemotherapy. Compliance was lower when the chemotherapy combination backbone was capecitabine instead of 5FU (*P* < 0.001).

**Table 2:** Chemotherapy adopted in first and in second line in the safety population (*n* = 225)

Chemotherapy	Patients, *N* (%)
First line in combination with cetuximab	
Irinotecan based	145 (64.4)
Oxaliplatin based	67 (29.8)
Other fluoropyrimidine based	13 (5.8)
Second line	119 (53.1)
Anti‐VEGF based	36 (31.2)
Oxaliplatin based	30 (25.2)
Irinotecan based	25 (21.0)
Fluoropyrimidine monotherapy	14 (11.8)
Anti‐EGFR based	9 (7.6)
Others	5 (4.2)

Median duration of cetuximab therapy, calculated as time between first dose and end of treatment, was 22.0 weeks (interquartile range: 10–36 weeks). Main reasons for treatment discontinuation were as follows: progression (45.8%), clinical deterioration without documented progression (10.2%), treatment toxicity (any grade, 8.4%), resection (7.1%), and death (4.9%). No cetuximab‐related deaths were reported. All enrolled patients had *KRAS* wild‐type mCRC. Mean time from *KRAS* test request to *KRAS* status determination was 13.53 days (SD = 13.1), median 10 days (range: 0–91).

### Quality of life

**Figure 2:**
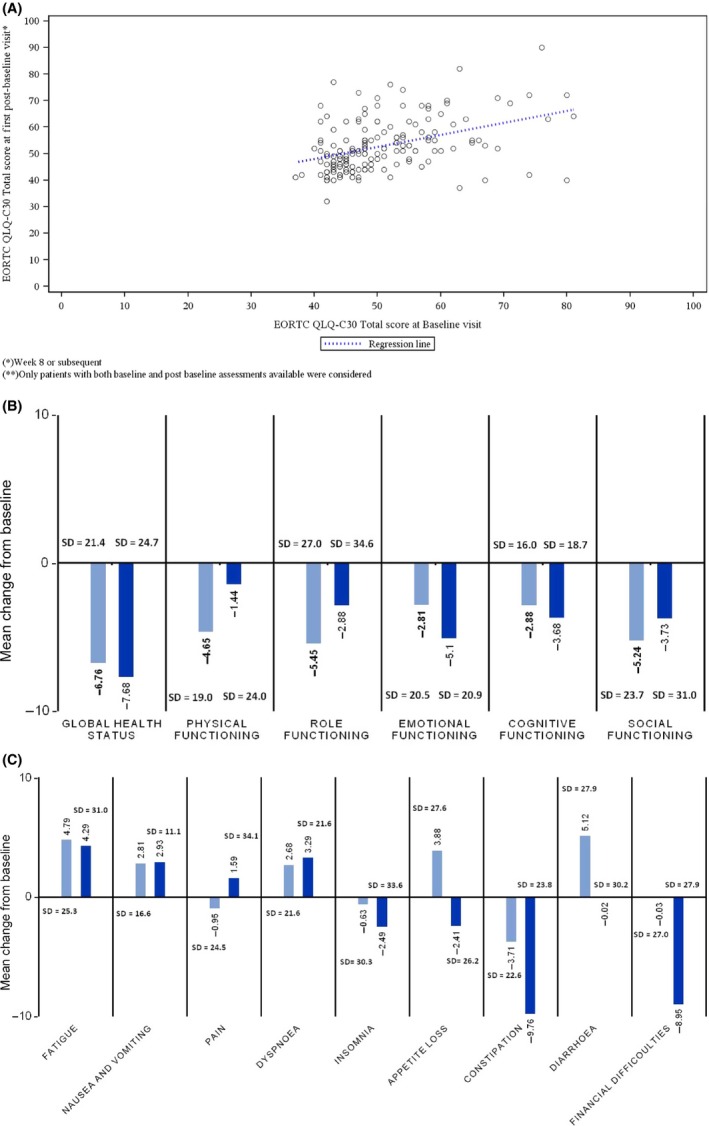
Results from EORTC QLQ‐C30 questionnaire for patients in the safety population with baseline and postbaseline assessments. (A) Association between total score at baseline and first postbaseline visit; dotted line indicates regression (*n *=* *158). (B) Mean change from baseline in Global Health Status and Function at first postbaseline visit (8–12 weeks—light blue; *n *=* *161) and at second postbaseline visit (20–24 weeks—dark blue; *n *=* *41). (C) Mean change from baseline for individual symptoms at first postbaseline visit (8–12 weeks—light blue; *n *=* *161) and to second postbaseline visit (20–24 weeks—dark blue; *n *=* *41); SD, standard deviation.

DLQI questionnaire results for both baseline and postbaseline assessments were available for 169 patients overall, as well as separately for skin management Protocols 1 and 2. The overall mean total DLQI score was 0.29 at baseline, and increased to 2.79 at the first postbaseline visit (mean change from baseline 2.50; SD = 4.138), which corresponds to a “small effect” (2–5 points) 15, 17. The mean values for each individual question ranged from 0 to 1, which corresponds to “no effect on dermatology‐related QoL” 15, 17. Figure 2A presents total scores on the EORTC QLQ‐C30 QoL questionnaire (*n *=* *158). A decrease in mean values was observed for all functional scales, including GHS/QoL (Fig. 2B), indicating slight worsening 18 during first‐line chemotherapy combined with cetuximab (*n *=* *161, first postbaseline evaluation; *n *=* *41 second postbaseline evaluation). No significant changes from baseline were recorded for individual symptoms (Fig. 2C; *n *=* *161, first postbaseline evaluation; *n *=* *41 second postbaseline evaluation).

### Secondary endpoints

Regarding the comparison between prophylactic and reactive skin management protocols, overall, the increase in DLQI score from baseline, and therefore a deterioration in QoL, was greater for patients receiving reactive compared to prophylactic treatment, but this was not significant (*P *=* *0.42). The only statistically significant difference was for the question “how itchy, sore, painful, or stinging has your skin been during the last week” (*P* = 0.0491). Again, reactive‐treated patients had a more impaired QoL than prophylactic‐treated patients, favoring the prophylactic skin management protocol (Table 3). The ANCOVA model confirmed the same trend for each item on the EORTC QLQ‐C30 (all *P *>* *0.05).

**Table 3:** Least square means of changes from baseline in the total DLQI score and the individual questions according to skin toxicity management protocols (safety set, *N* = 225)

Over the last week:	Least square mean (95% CI)		*P*‐value (ANCOVA model)
	Prophylactic (*N* = 160)	Reactive (*N* = 37)	
How itchy, sore, painful, or stinging has your skin been?	0.51 (0.37, 0.65)	0.83 (0.54, 1.11)	0.0491
How embarrassed or self‐conscious have you been because of your skin?	0.33 (0.21, 0.45)	0.52 (0.28, 0.76)	0.1663
How much has your skin interfered with you going shopping or looking after your home or garden?	0.23 (0.13, 0.33)	0.23 (0.03, 0.43)	0.9849
How much has your skin influenced the clothes you wear?	0.16 (0.07, 0.25)	0.26 (0.06, 0.45)	0.3703
How much has your skin affected any social or leisure activities?	0.18 (0.09, 0.27)	0.21 (0.02, 0.39)	0.7675
How much has your skin made it difficult for you to do any sport?	0.13 (0.05, 0.21)	0.06 (−0.1, 0.22)	0.4586
Has your skin prevented you from working or studying?	0.11 (0.01, 0.21)	−0.02 (−0.23, 0.18)	0.2687
If no, over the last week, how much has your skin been a problem at work or studying?	0.1 (0.04, 0.17)	0.24 (0.11, 0.38)	0.0708
How much has your skin created problems with your partner or any of your close friends or relatives?	0.18 (0.09, 0.27)	0.2 (0.02, 0.38)	0.8708
How much has your skin caused any sexual difficulties?	0.18 (0.08, 0.28)	0.17 (−0.03, 0.38)	0.9723
How much of a problem has the treatment for your skin been, for example, by making your home messy, or by taking up time?	0.16 (0.07, 0.24)	0.34 (0.16, 0.52)	0.065
Total score	2.34 (1.56, 3.11)	3.05 (1.48, 4.62)	0.4217

SD, standard deviation; DLQI, Dermatology Life Quality Index; CI, confidence interval.

Evaluating the EORTC QLQ‐C30 data with the ANOVA model revealed significant correlations between aspects of QoL and in particular previous primary tumor resection (Table 4), while there were no significant relationships between DLQI total score and predictors. Age did not affect any of the QoL items. AEs considered to be related to skin reactions were experienced by 176 patients (78.2%). There was no grade 4/5 skin AEs. Grade 3 AEs related to skin reactions occurred in 32 patients (14.2%). Significant relationships were found between the incidence of skin reactions and receiving an oxaliplatin combination (*P* < 0.0001) or male gender (*P* = 0.0008) (Table 5).

**Table 4:** Score change from baseline to first postbaseline visit on the EORTC QLQ‐C30 (*N* = 161)

	Global health status		Role functioning		Dyspnea		Diarrhea	
Predictors	Yes	No	Yes	No	Yes	No	Yes	No
Age ≥ 75 years	−5.8	−4.8	−0.7	−3.3	4.4	2.5	4.4	3.2
*P*	0.828		0.669		0.687		0.848	
Primary tumor surgery at baseline	−9.3	−1.3	−7.1	3.1	3.06	3.9	10	−2.3
*P*	0.049		0.047		0.838		0.021	
More than one metastatic site	−3.3	−7.3	1.8	−5.8	3.15	3.8	3.3	4.4
*P*	*P* = 0.252		0.087		0.852		0.808	
Irinotecan combination	−6.3	−4.3	−3.1	−0.9	7.22	−0.3	3.6	4.0
*P*	0.565		0.620		0.040		0.923	
Best response at first line	−3.8	−6.8	−1.2	−2.8	5.17	1.8	1.5	6.1
*P*	0.4		0.705		0.341		0.307	
Skin toxicity event in the first 8 weeks	−3.96	−6.63	−0.3	−3.7	1.9	5.1	1.5	6.2
*P*	0.54		0.533		0.467		0.410	
Overall model evaluation, *P*	0.268		0.188		0.493		0.259	

**Table 5:** Multivariate analysis of the incidence of cetuximab‐related skin reactions according to age, first‐line chemotherapy, and gender

Predictors	Incidence rate [×1000] (*N* = 212)	IRR (*N* = 212)	*P*
Age			
75+	23.47		
60−75	16.78	0.78 (0.61, 1)	0.0516
60	22.79	1.06 (0.83, 1.36)	0.6377
Chemotherapy in first line			
Irinotecan	16.3	1.72 (1.45, 2.04)	<0.0001
Oxaliplatin	26.98		
Gender			
Female	16	1.38 (1.14, 1.66)	0.0008
Male	21.85		

IRR (Incidence rate ratio) and respective *P*−value are estimated by multivariate Poisson regression.

Deaths during the study (*n* = 127) were attributed to tumors in 114 patients (90%). SAEs occurred in 53 patients (23.6%), mostly grade ≥3 (*n* = 45); the most common were intestinal obstruction (*n *=* *9); diarrhea (*n *=* *8); general physical health deterioration (*n *=* *5); hyponatremia, abdominal pain, and renal failure (*n *=* *3 each). In 12 patients (5.3%), the SAE resulted in death. A causal relationship with cetuximab was assessed for 12 patients (5.3%) with SAEs of grade 1–4. No cetuximab‐related deaths were recorded.

ORR was evaluated in 199 patients; no assessment of the best overall response was made for 26 (11.6%). Thirty‐one achieved a CR (13.8%) and 75 a PR (33.3%); 58 patients (25.8%) had stable disease; and 35 (15.6%) had progressive disease. The ORR (CR + PR) was 47.1%, and clinical benefit was 72.9%. In 45 patients (20.0%), ≥1 postbaseline surgical metastasis resection was reported, mainly from the liver (*n *=* *35). Median PFS was 8.3 months (95% CI: 6.70–9.40). After first‐line chemotherapy plus cetuximab, 119 patients (53.1% ‐ safety set) received second‐line chemotherapy. Considering only patients in progression of disease (*n* = 198), 109 patients (58, 1%) were treated with a second‐line chemotherapy.

**Figure 3:**
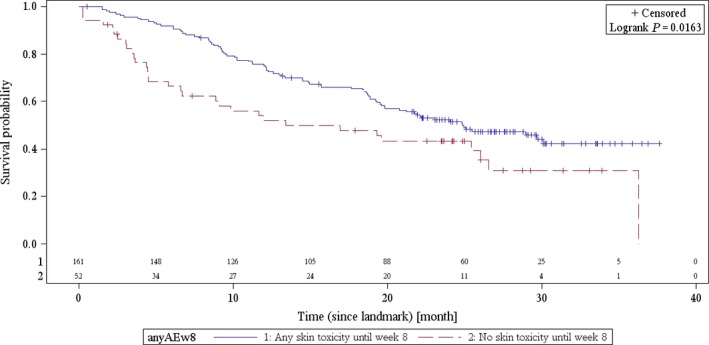
Overall survival by cetuximab‐related skin reaction (*N* = 225).

Median OS calculated for 225 patients was 23.6 months (95% CI: 20.47–27.27); ~50% of patients were still alive at 24 months. An exploratory landmark analysis of OS in subgroups defined by skin toxicity (any grade vs. no skin toxicity) showed a significant correlation between skin reaction events and superior overall survival (*P *=* *0.0163) (Fig. 3).

## Discussion

Cetuximab plus chemotherapy is standard first‐line treatment for *RAS* wild‐type mCRC 3, 8. However, the impact of cetuximab‐related skin reactions on patient QoL in routine practice first‐line setting have not been reported to date. To address this, we enrolled an unselected population in which about 40% of patients were over age 70 and more than half had comorbidities. The mean *KRAS* test turnaround time was within the 14‐day limit stipulated in the Italian guidelines 20. In first line irinotecan was administered more frequently than oxaliplatin in combination with cetuximab, both combinations were safe and did not affect compliance.

Results with the DLQI and EORTC QLQ‐C30 questionnaires suggest that cetuximab plus chemotherapy does not have a negative impact on QoL in patients treated in routine practice with first‐line therapy for mCRC, supporting the results from the controlled CRYSTAL trial 9. We sought predictors of lower QoL through multivariate analysis and, while skin reactions were not significant, other factors were correlated with lower QoL in this setting. Of interest previous surgery for primary tumors at baseline predicted reduced QoL in terms of GHS, Role Functioning and Diarrhea on the EORTC QLQ‐C30 (Table 4), suggesting that patients would benefit from better supportive care in daily practice, to limit uneasiness and discomfort after primary tumor resection.

Both prophylactic and reactive skin management protocols produced similar favorable QoL outcomes, suggesting that careful management of skin toxicity with either of these protocols can limit the negative impact of this side‐effect on QoL in routine clinical practice and favor treatment compliance. The small number of patients using Skin Protocol 3 precluded analysis of outcomes.

Although no differences were found between the two protocols, the ObservEr investigators preferred the prophylactic protocol, because the patients found the added attention reassuring.

We observed the same correlation between OS and skin reactions that was reported in controlled clinical trials, and here too these adverse events were associated positively with efficacy. Discussing this correlation with patients should be considered when providing psychological support during therapy 21. Regarding efficacy, the median OS of 24 months is in line with findings from randomized trials in first‐line in patients with *KRAS* wild‐type tumors 1, 2, as were ORR, mRR, and PFS, the limitations of an observational study notwithstanding. Only about half of patients were amenable to second‐line therapy, indicating that in routine practice first line therapy is the most important component of an mCRC strategy 22, 23.

In the safety analysis, only 14% of patients experienced a grade ≥3 skin reaction and there were no cetuximab‐related deaths. These reporting frequencies suggest a good safety profile for cetuximab 1, 4, 5, despite the likelihood that unselected patients in an observational study tend to be in poorer general condition and to have lower tolerance for treatment than those selected for clinical trials.

In conclusion, in this observational experience chemotherapy plus cetuximab achieved the same effectiveness seen in randomized trials 1, 2, 5. The availability of management recommendations 14 improved safety of cetuximab with limited severe skin‐related AEs and preserved compliance with treatment. The ObservEr study demonstrates that cetuximab‐based therapy in an unselected “real world” population can be effective without having a negative impact on QoL. We observed the same correlation between OS and skin reactions that was reported in controlled clinical trials, and here too these adverse events were associated positively with efficacy 24, 25, 26, 27.

## Conflicts of Interest

None declared.

## Clinical Investigators

Vincenzo Adamo, A.U. G. Martino, Messina; Oscar Alabiso, Ospedale Maggiore della Carità Novara; Antonio Ardizzoia, Ospedale A. Manzoni, Lecco; Antonio Bernardo, Fondazione Maugeri, Pavia; Domenico Bilancia e Gerardo Rosati, Ospedale San Carlo, Potenza; Silvana Chiara, Istituto Nazionale per la Ricerca sul Cancro, Genova; Libero Ciuffredda, A.O. Città della Salute e della Scienza, Le Molinette, Torino; Mario Clerico, Ospedale degli Infermi, Biella; Francesco Di Costanzo and Lorenzo Antonuzzo, Azienda Ospedaliero Universitaria Careggi, Firenze; Francesca Di Fabio e Carmine Pinto, Policlinico Sant'Orsola Malpighi, Bologna; Raimondo Ferrara, Ospedale Mons. R. Dimiccoli, Barletta, Bari; Daris Ferrari, A.O. San Paolo, Milano; Francesco Ferraù, Ospedale San Vincenzo, Taormina, Messina; Antonio Frassoldati, A.O. U. Arcispedale Sant'Anna, Ferrara; Carmelo Iacono, Ospedale Maria Paternò Arezzo, Ragusa; Giovanni Pietro Ianniello, A.O. Sant'Anna e San Sebastiano, Caserta; Ivan Roberto Lolli, IRCCS Saverio De Bellis, Castellana Grotte, Bari; Giovanni Lo Re, A.O. Santa Maria degli Angeli, Pordenone; Rodolfo Mattioli, Ospedale Santa Croce, Fano, Pesaro; Mauro Minelli, Ospedale Sant'Eugenio, Roma; Lorenzo Pavesi, Fondazione Maugeri, Pavia; Graziella Pinotti, Ospedale Circolo e Fondazione Macchi, Varese; Giovanni Rosti, Ospedale Santa Maria di Ca'Foncello, Treviso; Enzo Maria Ruggeri, Ospedale di Belcolle, Viterbo; Paolo Tralongo, Ospedale “Umberto I” RAO Siracusa (SR); Michele Valeri, Ospedale Civile di Macerata, Macerata; Giovanni Vicario, Ospedale San Giacomo Apostolo, Castel Franco Veneto; Guido Giacomo Vietti Ramus, Ospedale Torino Nord San Giovanni Bosco, Torino; Germano Zampa, Ospedale Nuovo Regina Margherita, Roma.
